# Phone It In: A Medical Student Primer on Telemedicine Consultation in Pediatrics

**DOI:** 10.15766/mep_2374-8265.11067

**Published:** 2021-01-07

**Authors:** Lauren M. McDaniel, Matthew Molloy, Daniel J. Hindman, Suzanne R. Kochis, W. Christopher Golden, Amit K. Pahwa, Tina Kumra

**Affiliations:** 1 Chief Resident, Department of Pediatrics, Johns Hopkins University School of Medicine Baltimore; 2 Assistant Professor, Departments of Medicine and Pediatrics, Johns Hopkins University School of Medicine Baltimore; 3 Fellow in Allergy and Immunology, Division of Allergy and Immunology, Department of Pediatrics, Johns Hopkins University School of Medicine Baltimore; 4 Associate Professor, Department of Pediatrics, Johns Hopkins University School of Medicine Baltimore; Medical Director, Newborn Nursery, Johns Hopkins University School of Medicine Baltimore; Director, Pediatric Core Clerkship, Johns Hopkins University School of Medicine Baltimore; 5 Assistant Professor, Departments of Medicine and Pediatrics, Johns Hopkins University School of Medicine Baltimore; Director of Internal Medicine Subinternship and Assistant Director of Pediatric Core Clerkship, Johns Hopkins University School of Medicine Baltimore; 6 Assistant Professor, Department of Pediatrics, Johns Hopkins University School of Medicine Baltimore; Medical Director, Johns Hopkins Community Physicians at Remington; Clinical Director, Bloomberg School of Public Health, General Preventative Medicine Residency

**Keywords:** Telephone, Triage, Telemedicine, Telehealth, Consultation, Remote Consultation

## Abstract

**Introduction:**

Telephone triage requires a unique skillset that is not universally taught in medical school. This curriculum was developed to introduce third- and fourth-year medical students participating in their pediatrics core clerkship to the benefits, challenges, and mechanics of telephone triage.

**Methods:**

After completing a presession textbook reading and listening to a brief lecture, students participated in two telephone role-play scenarios with parents. The exercise required students to recognize the differences in acuity level of patients and provide appropriate guidance, management, and disposition instructions. Following the session, students completed a telephone note. Students evaluated this curriculum at the completion of the clerkship.

**Results:**

The majority of the 74 students who completed the 5-point Likert scale evaluation felt that the curriculum met its stated objectives (a score of 4 or 5 given by 82%), increased their knowledge (73%), engaged them (86%), and was of high quality (82%). Students specifically commented that the experience was useful, interactive, and applicable to their clerkship experience and future career. The most common area of constructive feedback was not understanding the purpose of a telephone note.

**Discussion:**

This easily implemented curriculum provided a foundational experience in the nuances of triaging and managing pediatric patients via the telephone. This serves as an important framework to prepare students for more complex telemedicine technology.

## Educational Objectives

By the end of this activity, learners will be able to:

1. List questions to ask a patient or patient's caregiver who calls a triage line based on the patient's chief concern.
2. Differentiate severity of illness through phone conversation with a patient or patient's caregiver.
3. Develop a management plan based on assessment through a phone conversation.

## Introduction

Patients, physicians, and health insurers have recognized the value of telephone triage services for cost-saving, convenience, and decreasing unnecessary use of the health care system.^1^ Despite these advantages, telephone triage presents challenges to the provider, who inherently is presented with less data than an in-person encounter (most notably, a complete physical exam). Learning to efficiently and effectively gather data, triage based on patient acuity, and provide recommendations to patients and families, all while physically separated from a patient, becomes a crucial skillset for providers to master. Despite the importance of this skillset, many providers during and after residency feel unprepared to manage patients via the telephone.^2,3^ A recent Cochrane review urgently called for additional research and training on telephone consultation skills.^4^

Our objective was to address this gap in medical education by developing a curriculum targeted at medical students based in Kolb's experiential learning theory.^5^ Kolb's method aims to provide concrete experiences practicing a skill, allow for reflective observation, and then ask learners to apply what they have learned to additional experiences through active experimentation. Modeling this theory, we developed a curriculum which involved the application of presession and didactic learning to two practice role-play cases, during and in between which students were provided feedback and additional teaching points.

The clinical reasoning skills that are developed in the practice of telephone triage can be applied to in-person encounters as well. Students learn to differentiate children who are sick from those who are well and are given the opportunity to practice anticipatory guidance, a skillset that is important in all patient encounters. With the limited information available during phone triage, clinical reasoning skills and expansion of differential diagnoses outside the realm of common diagnoses are required in order to consider what otherwise may be easily missed without further in-person evaluation. These clinical thinking skills are particularly critical for third- and fourth-year medical students to practice experientially using role-play. The utility of these skills continues beyond medical school training. While registered nurses take the majority of these triage calls, physicians are relied upon to review and approve dispositions recommended and to make decisions when presentations are not clear.

To our knowledge, no curriculum to date has been published that educates medical students on their pediatric core clerkship about telephone triage. While published curricula on this topic is limited, others have utilized Kolb's experiential learning theory to develop standardized patient video-visit cases for clerkship students to practice remotely treating adult patients in a rural setting.^6^ Similarly, a telephone triage curriculum given to pediatric residents has been shown to improve history taking and management.^7^ In designing this curriculum, we felt that it was critical for the learner to feel comfortable with the mechanics of telephone triage, the most frequently accessed telehealth technology.^1^ Our goal was to create a curriculum that was both meaningful, but also minimally resource-intensive, to specifically challenge learners to triage pediatric patients they could not visualize and to whom they could not speak.

## Methods

We designed our curriculum using the six-step approach outlined in the *Curriculum Development for Medical Education: A Six-Step Approach*.^8^ This curriculum and associated evaluation were introduced into the pediatric core clerkship in August of 2017, with evaluations of the curriculum beginning in January of 2018. Once every academic quarter, 20–25 third- and fourth-year students received this 1-hour training as part of their scheduled core pediatrics clerkship educational sessions. Students participating in the curriculum had some experience conducting a history. One faculty member and one pediatrics resident, chief resident, or fellow facilitated the sessions. Facilitators had experience with pediatric phone triage in order to most accurately improvise when students’ questions covered topics outside of the case script. The location of this workshop was a room with the ability to view PowerPoint presentations and had access to both cellular and landline phone services. Two different telephones were utilized in this exercise. The facilitator guide (Appendix A) can be used to guide the presentation and phone triage exercise, particularly with respect to timing and debrief teaching points.

Prior to the session, we provided students with the text of a page they would receive on a pager as the on-call physician during this session from a parent requesting a callback (this page can be found on the final slide of Appendix B). This page read: “13-month-old female. Cough. Fever. Poor appetite. Please call.” Students were also assigned textbook prereading material on conducting telephone interviews for the chief complaints of cold and cough^9^ and were encouraged to brainstorm questions they would like to ask the parent. At the beginning of the workshop, one facilitator presented a brief, 10-minute PowerPoint presentation (Appendix B; speaker notes can be found in Appendix C) to introduce the students to the concept of telephone triage, highlighting the importance of this skillset, potential drawbacks associated with the use of this technology, and the general structure and decision-making process involved in telephone triage phone calls.

One student volunteer was then asked to call (on speakerphone) the parent of the child referenced in the preassigned page. At this time, one facilitator stepped outside of the lecture room to act the part of the parent by answering the call of the student. This facilitator utilized the case example (Appendix D) to answer the student's questions. The student was prompted by the facilitator remaining in the room to take time-outs during this phone call to consult their colleagues about additional questions to ask the parent and to discuss the appropriate disposition of the child. At the end of the first case, the second facilitator returned to the room to debrief the case and provide students with feedback. The facilitators then switched roles, and a new student proceeded with the second phone triage case (Appendix D).

The first case represented a child with mild viral symptoms who could safely remain home with reassurance, management guidance, and return precautions, while the second case detailed a more acute presentation of increased work of breathing and dehydration necessitating urgent evaluation at an emergency department. During the time-outs, the facilitator prompted the students to think about how one might assess work of breathing and hydration status without being able to visualize the child. The students were challenged to reflect on which questions helped them to most efficiently determine whether a child required an in-person evaluation. If the student neglected to provide a critical instruction (e.g., when the parent should call back), then the facilitator would prompt the student with a reminder. After the completion of the session, the students were asked to document their phone encounter as they would in a medical record. Once submitted, the students were provided feedback on these notes.

At the end of the 8-week pediatrics clerkship, we surveyed the students about the session using the same instrument that that was distributed for all lectures in the pediatric clerkship. The relevant evaluation questions to this session (Appendix E) included four questions using a 5-point Likert scale. The questions asked students to evaluate whether the session met the stated objectives, whether it increased their knowledge of the topic, whether the presenter's presentation style actively engaged them, and to rate the overall quality of the session with 5 representing *completely*, *strongly agree*, or *excellent*. Participant comments were reviewed manually and organized into themes. Comments that referenced a different session or that did not include feedback (e.g., “thanks”) were excluded from thematic analysis.

The curriculum and evaluation were deemed exempt by the Johns Hopkins Institutional Review Board (IRB00212520).

## Results

A total of 171 students participated in the educational session. Of these, 74 (43%) responded to the postsession survey, which utilized a 5-point Likert scale (Appendix E). Of the students who responded, 61 (82%) of students responded with a 4 or 5 (5 = *completely*) to the question, “Did the seminar meet the stated objectives?” Of students, 68 (92%) felt that the seminar at least somewhat increased their knowledge of the topic, with 54 (73%) giving a 4 or 5 (5 = *strongly agree*). Of students, 64 (86%) responded to the statement, “The presenter's style actively engaged me,” with a 4 or 5 (5 = *strongly agree*). When asked to rate the overall quality of the session, 61 students (82%) rated it a 4 or 5 (5 = *excellent*).

Thematic analysis of the 16 student comments revealed three common themes—relevance, engagement, and desire for more information. The first theme was generic feelings about the utility and relevance of the exercise. Five students commented that they felt the session was “useful,” “helpful,” or “practical.” One student commented, “Useful session on a skill that we don't often hear about or get to practice. I think it's a very practical session for what the future practice of medicine might look like.” Another stated, “Great and helpful topic as I'm being exposed to more phone medicine on the rotation. Helpful to put a structure to this new activity.” Another noted that they now had an “…appreciation that telephone triage is difficult.” The second theme involved comments on participants’ level of enjoyment of and engagement in the session. Three students specifically noted that the session was “interactive” and “engaging,” while two others noted that they “enjoyed the calling exercise.” One commented specifically, “Great interactive session, and I liked that even though there were just two volunteers who got to do the phone call, we could chime in with added questions and suggestions.” Common themes of constructive feedback related to not completely understanding the utility of the phone note write-up at the end of the session with three students commenting on this and one requesting a sample phone note write-up. Two students requested more time spent on reviewing key questions to ask on a phone interview.

## Discussion

As we enter a new era of medicine particularly with respect to the recent COVID-19 pandemic, proficiency in telephone triage has never been more important. Even as technology advances, the telephone remains a key tool for patients and families to access the health care system. Early in their training, medical students and residents return calls of clinic patients and follow-up on hospitalized patients postdischarge. Soliciting and providing information via a telephone requires a different approach and skillset than the traditional history and physical exam. A prior study of this curriculum at our institution demonstrated that dedicated medical student education in telephone medicine improved value of care by decreasing the number of nonindicated tests ordered by the students.^10^

This curriculum was designed to introduce medical students to the nuances and challenges of telephone triage. Our results suggested that structured facilitated role-play is an easy, effective, and inexpensive format. This exercise allowed students to practice developing rapport and trust without in-person contact. They gained experience verbalizing which historical and physical signs are suggestive of an ill or decompensating patient. Unable to rely on their own physical exam, they learned the important skill of teaching patients and family members how to elicit and report important findings. This exercise additionally provided the student with experience delivering anticipatory guidance and return-to-care instructions.

We believe the simplicity and reproducibility of this curriculum are of great value to educators. While requiring minimal time and resources, it effectively and efficiently simulated the challenges of telephone triage and allowed students to refine their data-gathering and synthesis skills. The time-outs served as opportunities to integrate reflective observation, a critical part of Kolb's learning cycle.^5^ Overall, students valued this experience and evaluated it favorably. The student comments following the exercise highlighted the usefulness and applicability of the curriculum and the interactive format. They appreciated the deviance from the traditional lecture model and recognized the relevance of this exercise to their future careers. Feedback from students indicated a desire for more didactic introduction to the structure of telephone triage and subsequent documentation. Time, of course, was an inherent challenge to this, as expanding the lecture portion of this curriculum would be at the expense of practicing and role-playing within the exercise. This balance can and should be refined based on the learner level and learning environment as this material is adopted elsewhere. Based on our experience, we would recommend providing students with a brief, verbal introduction to the structure and importance of telephone note documentation, as several students expressed confusion about the relevance and applicability of this aspect of the exercise. Future iterations of this curriculum would benefit from inclusion of more discussion regarding appropriate documentation of the phone encounter and provide examples of this documentation.

Our curriculum was limited to telephone technology. As telehealth resources expand, providers will have increasing access to remote visualization and examination of patients. Nonetheless, the telephone remains the most widely utilized telemedicine technology. We felt it was important for the focus of this exercise to be on the skills of triage and consultation, rather than mastery of the technology itself. Our results were subject to many of the biases inherent in surveys, most notably nonresponse bias, as many students who participated in the curriculum did not complete the end-of-rotation evaluations. Additionally, we did not stratify students based on their year in training, their specific pediatric rotation experiences (e.g., clinic, emergency department, community/academic hospital), or their career intentions, which may have affected students’ baseline level of knowledge and immediate applicability of the curriculum. Our survey design could be improved in future uses by labeling all response options, not just 1, 3, and 5. Finally, it is important to note that the measure we studied was medical student reactions to the curriculum. Although we did ask students if the seminar met the stated learning objectives, we did not specifically evaluate these objectives. Additional work can and should evaluate higher levels of evidence in the Kirkpatrick model of training evaluation.^11^ This could include demonstration of learning (e.g., a quiz), behavior (e.g., performance during a simulated case), or results (e.g., application of skills to real clinical encounters).

Additional literature and curricula are needed at the medical student, resident, and postresidency levels to establish best practices in the use and adaptation of each emerging technology in health care. We hope that this curriculum can serve as a foundational course in telephone triage. As remote care becomes more available, there will be an increased need for telemedicine triage education to include other digital formats including virtual visits and asynchronous messaging. Our curriculum allows for the early introduction of a framework for these encounters in the training of future physicians, who inevitably will spend the remainder of their careers adapting to rapidly advancing telehealth technologies.

## Appendices

Facilitator Guide.docxPhone It In Presentation.pptxSpeaker Notes.docxTelemedicine Cases.docxSession Evaluation.docx All appendices are peer reviewed as integral parts of the Original Publication.
