# Supplementary material for: Phone It In: A Medical Student Primer on Telemedicine Consultation in Pediatrics
Source: MedEdPORTAL. 2021 Jan 7;17:11067. doi: 10.15766/mep_2374-8265.11067 (PMC7809927; doi:10.15766/mep_2374-8265.11067)
Supplement: Supplementary file 1 — Facilitator Guide.docxPhone It In Presentation.pptxSpeaker Notes.docxTelemedicine Cases.docxSession Evaluation.docx [file mep_2374-8265.11067-s001.zip › A. Facilitator Guide.docx]

**Facilitator Guide**

Venue: Lecture room

Facilitators: 1 faculty member and one resident/fellow/additional faculty member

Materials: Computer and projector for presentation and two telephones (landline or cellular)

**Session layout**

| **Activity** | **Time** |
| --- | --- |
| Introduction | 5 minutes |
| Core didactics | 10 minutes |
| Telephone case #1 | 15 minutes |
| Debrief | 5 minutes |
| Telephone case #2 | 15 minutes |
| Wrap up | 5 minutes |

**Practical instructions:**

1. Introductions

Familiarize yourself with the level of telephone triage experience among students.

1. Core didactics

Use the PowerPoint provided or a similar slide deck to present the importance of telephone triage, potential drawbacks associated with the use of this technology, and the general structure and decision-making process involved in these phone calls.

1. Telephone case #1

One student volunteer comes to the front of the room and utilizing the phone on speaker, returns the call of the parent. One facilitator leaves the lecture room to answer call and act the part of the parent. Remaining facilitator in the room directs the use of “time outs” to ensure case progression, group participation, and to make relevant teaching points. The first case represents a child with mild viral symptoms who can safely remain home with reassurance, management guidance, and return precautions. The child has no increased work of breathing, no evidence of dehydration, and has normal activity levels. At the end of this case, the facilitator should ensure that the student has instructed the parent on return-to-care instructions and that the student has ensured parental understanding of these recommendations.

1. Debrief

Ask the student who led the case to reflect on what went well and what was challenging. The other students and the facilitators can also provide feedback. If not already discussed during the case, facilitators should prompt students to reflect on which questions helped them to most efficiently determine whether a child required an in-person evaluation.

Facilitators should highlight that changes in work of breathing, hydration, or mental status warrant urgent evaluation and students should focus their questions around these topics. Facilitators should review questions that help to assess for these changes. Students should ask parents to take off the child’s shirt and to look for pulling of muscles underneath the child’s rib cage, in between the child’s ribs, or around the child’s neck. They should also ask the parent about flaring of the child’s nostrils or bobbing of the child’s head with breathing. Students may ask the parent to count the child’s respiratory rate for one minute. They may ask if the child seems out of breath with activity or if the child seems more fatigued or difficult to awaken than usual. To assess dehydration, students should ask for details about the volume of liquid consumed and the frequency/amount of urine output. If the child does not have concerning findings at the time of telephone triage, the student should advise the parent to watch for these symptoms.

1. Telephone case #2

Facilitators switch roles and a second student should proceed with the second phone triage case. The second case details a more acute presentation of increased work of breathing and dehydration necessitating urgent evaluation at an emergency department. At the end of this case, the facilitator should ensure that the student has instructed the parent to either take the child to the nearest ED or to call 9-1-1. The student should confirm parental understanding. The facilitator could also choose to discuss how the student might call the ED to let them know that the child is coming and to ask the ED provider to let the student know when the child has arrived.

1. Wrap up

Allow students to reflect on what they learned from the session and ask remaining questions. Introduce the post-session telephone note exercise, detailing structure and importance of these notes. The facilitator should highlight how these notes are important for communication to other providers, assurance of appropriate follow-up, medico-legal purposes, and for reimbursement.
