# Supplementary material for: Phone It In: A Medical Student Primer on Telemedicine Consultation in Pediatrics
Source: MedEdPORTAL. 2021 Jan 7;17:11067. doi: 10.15766/mep_2374-8265.11067 (PMC7809927; doi:10.15766/mep_2374-8265.11067)
Supplement: Supplementary file 1 — Facilitator Guide.docxPhone It In Presentation.pptxSpeaker Notes.docxTelemedicine Cases.docxSession Evaluation.docx [file mep_2374-8265.11067-s001.zip › B. Phone It In Presentation.pptx]

## Slide 1
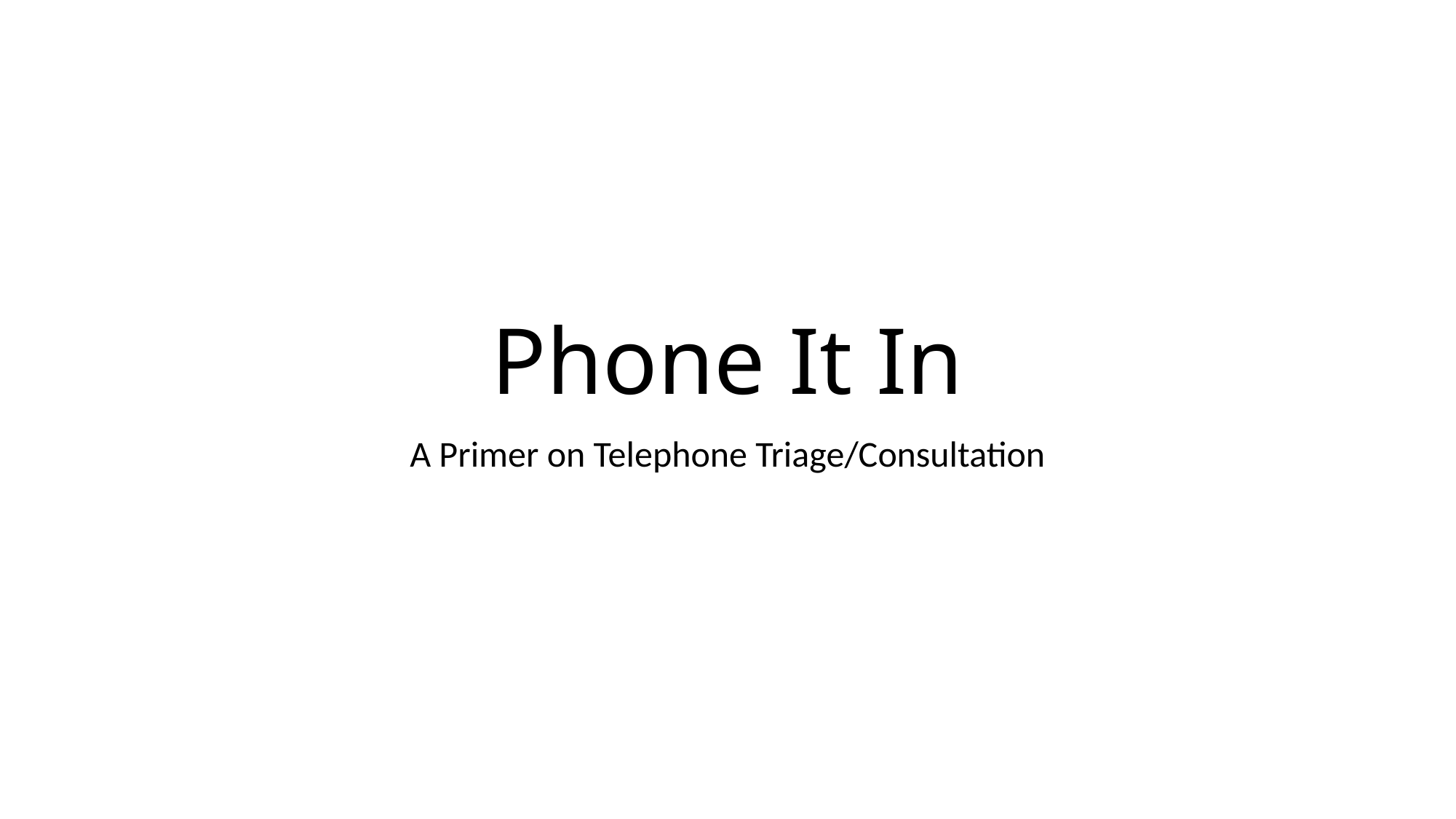

# Phone It In
A Primer on Telephone Triage/Consultation

## Slide 2
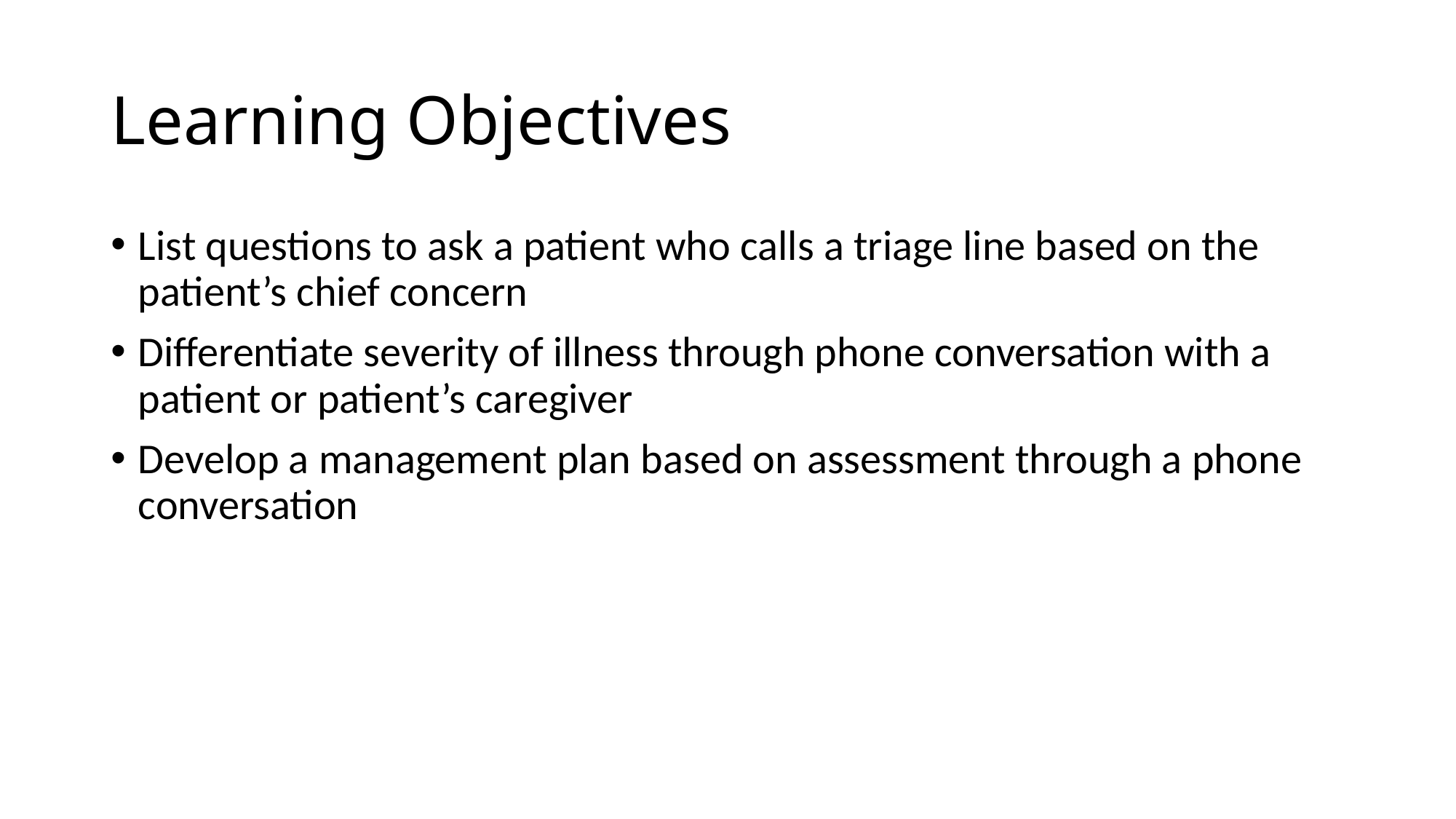

# Learning Objectives
List questions to ask a patient who calls a triage line based on the patient’s chief concern
Differentiate severity of illness through phone conversation with a patient or patient’s caregiver
Develop a management plan based on assessment through a phone conversation

## Slide 3
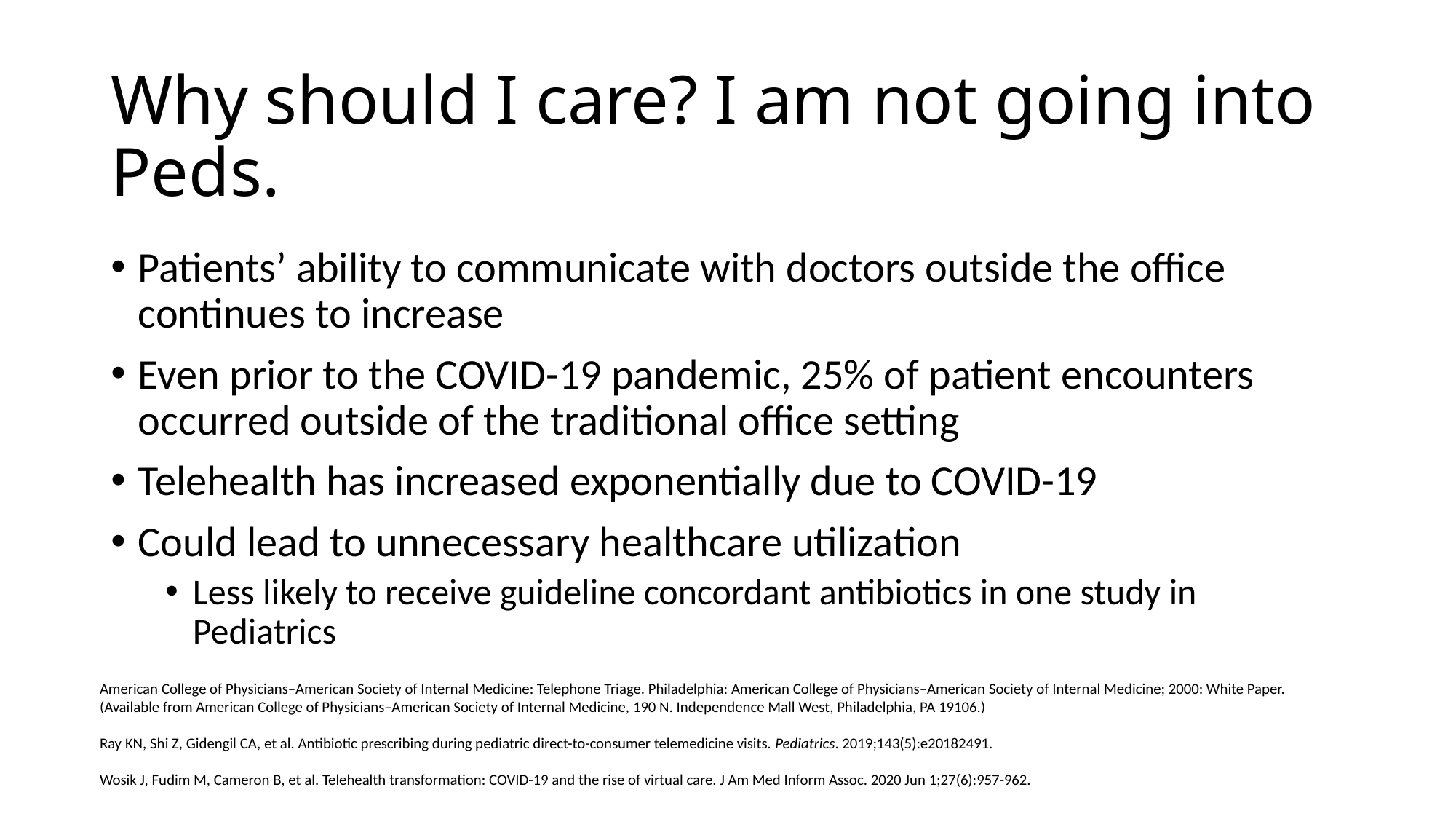

# Why should I care? I am not going into Peds.
Patients’ ability to communicate with doctors outside the office continues to increase
Even prior to the COVID-19 pandemic, 25% of patient encounters occurred outside of the traditional office setting
Telehealth has increased exponentially due to COVID-19
Could lead to unnecessary healthcare utilization
Less likely to receive guideline concordant antibiotics in one study in Pediatrics
American College of Physicians–American Society of Internal Medicine: Telephone Triage. Philadelphia: American College of Physicians–American Society of Internal Medicine; 2000: White Paper. (Available from American College of Physicians–American Society of Internal Medicine, 190 N. Independence Mall West, Philadelphia, PA 19106.)
Ray KN, Shi Z, Gidengil CA, et al. Antibiotic prescribing during pediatric direct-to-consumer telemedicine visits. Pediatrics. 2019;143(5):e20182491.
Wosik J, Fudim M, Cameron B, et al. Telehealth transformation: COVID-19 and the rise of virtual care. J Am Med Inform Assoc. 2020 Jun 1;27(6):957-962.

## Slide 4
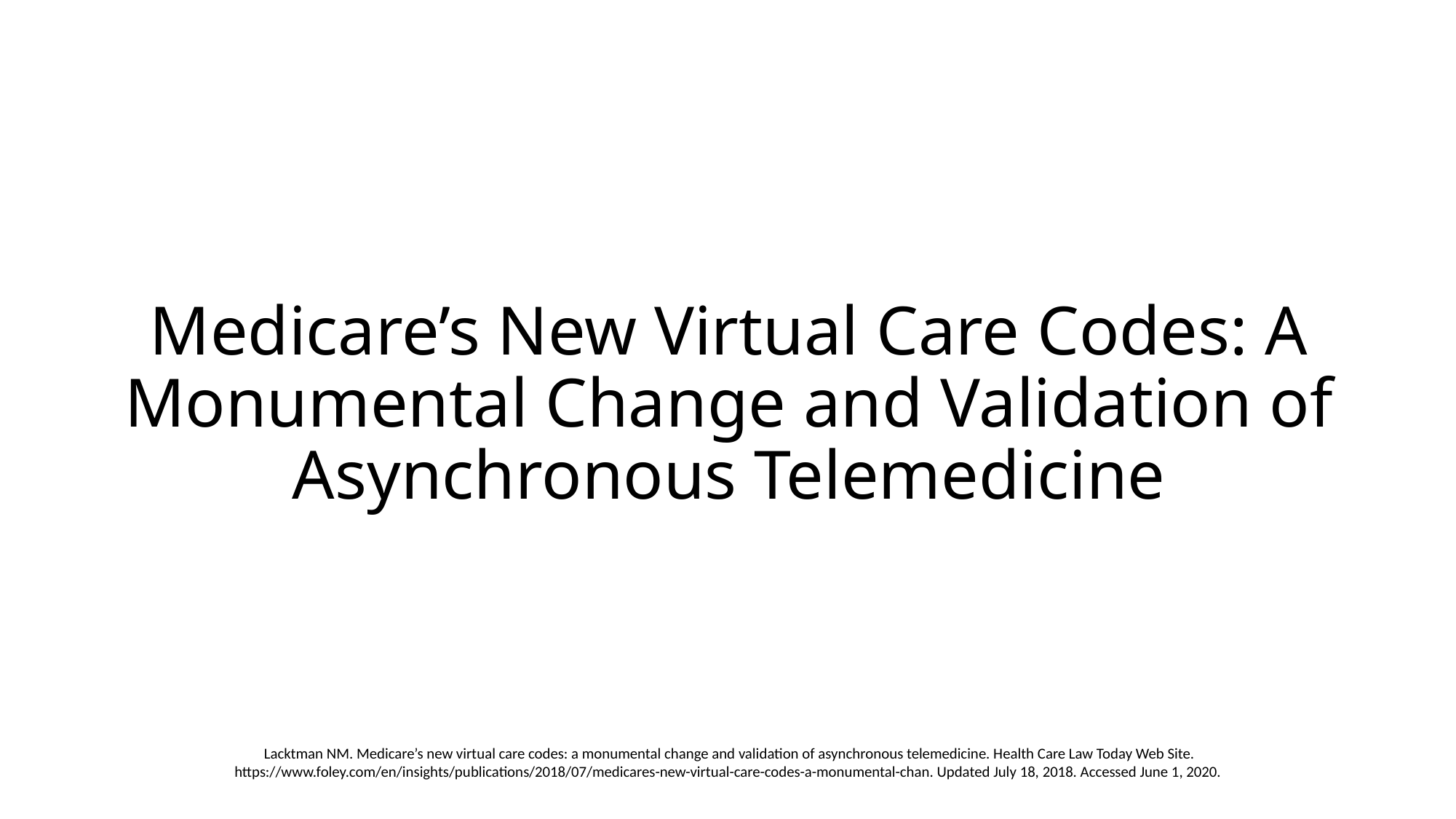

# Medicare’s New Virtual Care Codes: A Monumental Change and Validation of Asynchronous Telemedicine
Lacktman NM. Medicare’s new virtual care codes: a monumental change and validation of asynchronous telemedicine. Health Care Law Today Web Site. https://www.foley.com/en/insights/publications/2018/07/medicares-new-virtual-care-codes-a-monumental-chan. Updated July 18, 2018. Accessed June 1, 2020.

## Slide 5
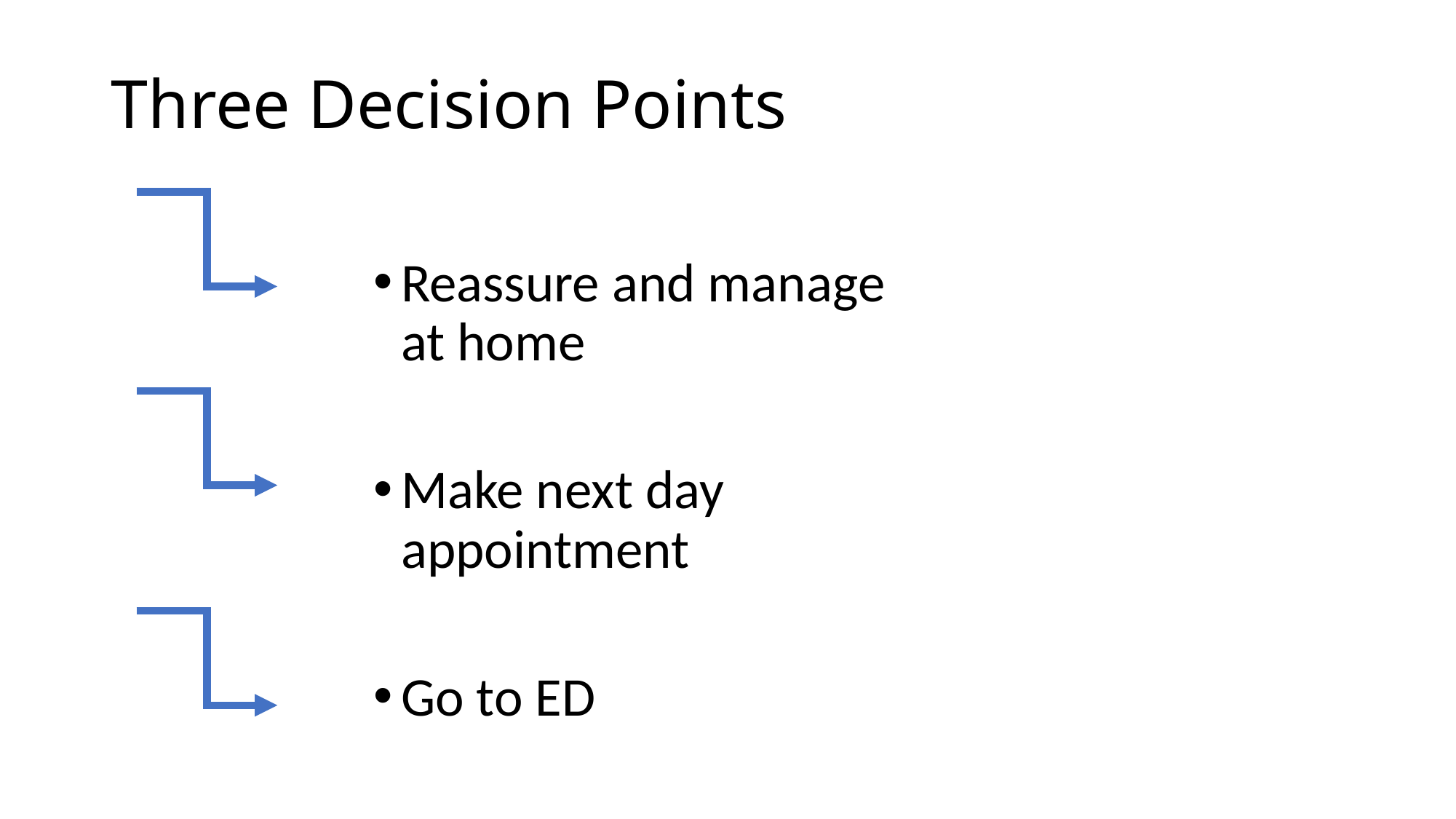

# Three Decision Points
Reassure and manage at home
Make next day appointment
Go to ED

## Slide 6
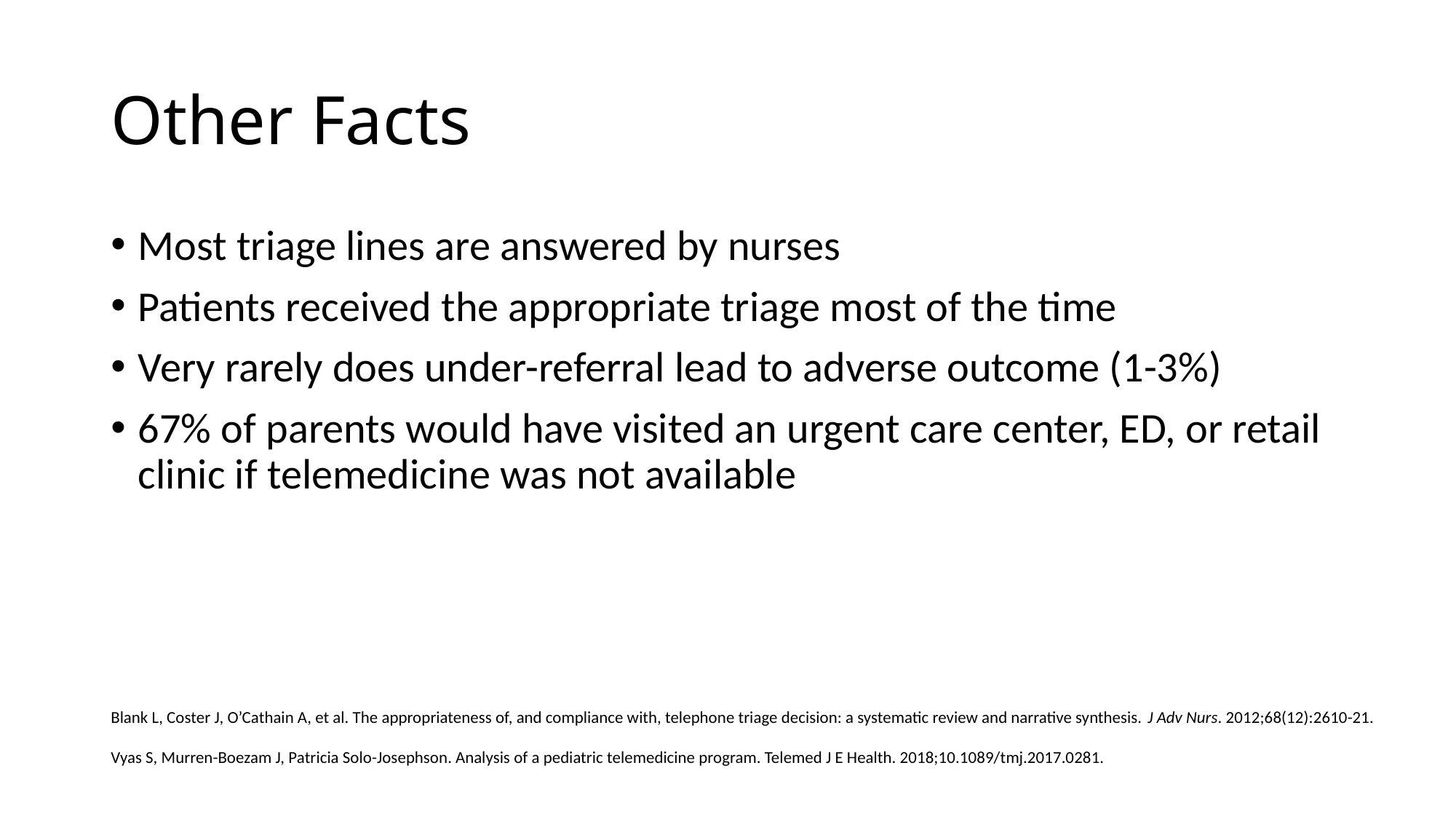

# Other Facts
Most triage lines are answered by nurses
Patients received the appropriate triage most of the time
Very rarely does under-referral lead to adverse outcome (1-3%)
67% of parents would have visited an urgent care center, ED, or retail clinic if telemedicine was not available
Blank L, Coster J, O’Cathain A, et al. The appropriateness of, and compliance with, telephone triage decision: a systematic review and narrative synthesis. J Adv Nurs. 2012;68(12):2610-21.
Vyas S, Murren-Boezam J, Patricia Solo-Josephson. Analysis of a pediatric telemedicine program. Telemed J E Health. 2018;10.1089/tmj.2017.0281.

## Slide 7
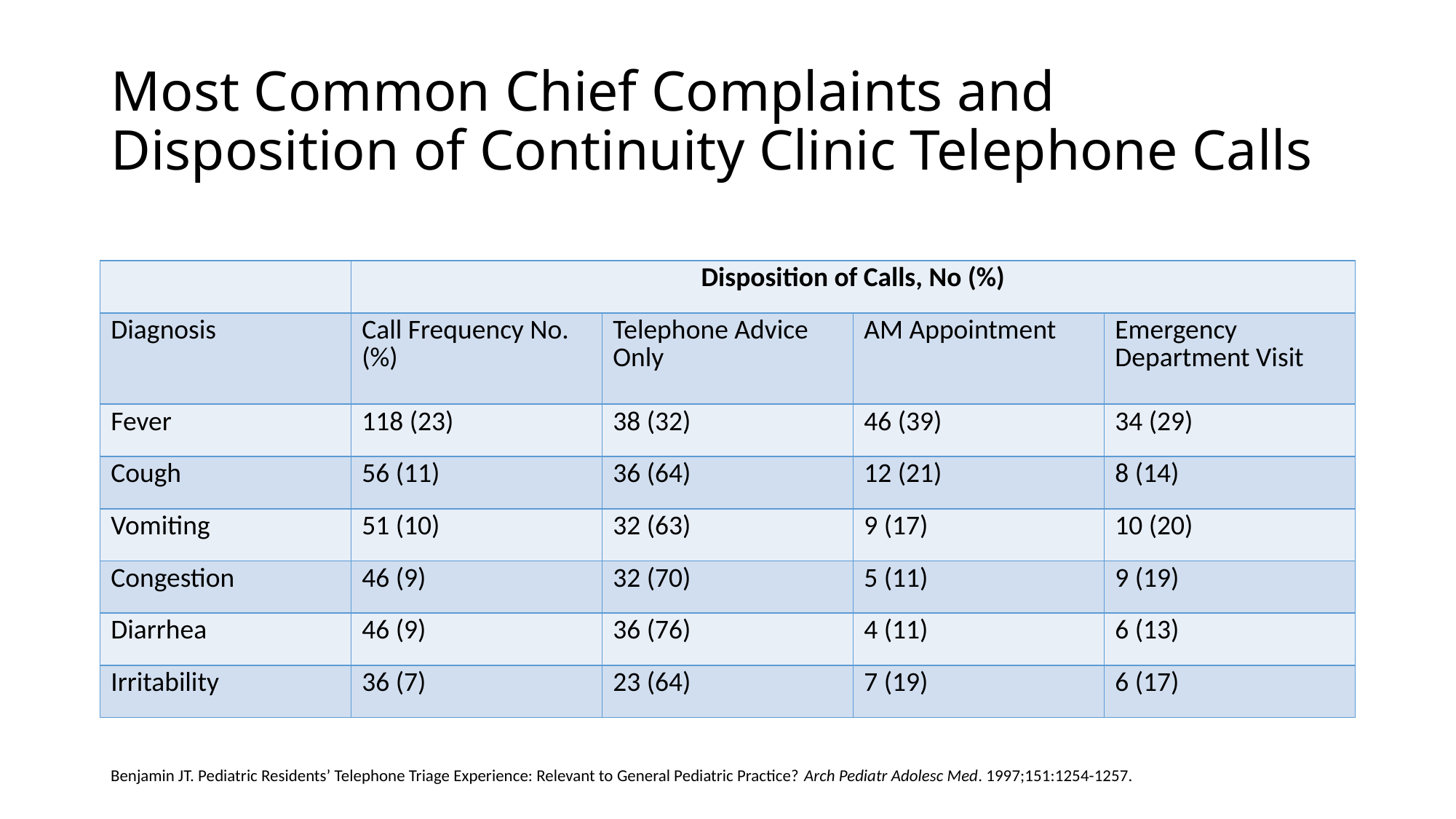

# Most Common Chief Complaints and Disposition of Continuity Clinic Telephone Calls
| | Disposition of Calls, No (%) | | | |
| --- | --- | --- | --- | --- |
| Diagnosis | Call Frequency No. (%) | Telephone Advice Only | AM Appointment | Emergency Department Visit |
| Fever | 118 (23) | 38 (32) | 46 (39) | 34 (29) |
| Cough | 56 (11) | 36 (64) | 12 (21) | 8 (14) |
| Vomiting | 51 (10) | 32 (63) | 9 (17) | 10 (20) |
| Congestion | 46 (9) | 32 (70) | 5 (11) | 9 (19) |
| Diarrhea | 46 (9) | 36 (76) | 4 (11) | 6 (13) |
| Irritability | 36 (7) | 23 (64) | 7 (19) | 6 (17) |
Benjamin JT. Pediatric Residents’ Telephone Triage Experience: Relevant to General Pediatric Practice? Arch Pediatr Adolesc Med. 1997;151:1254-1257.

## Slide 8
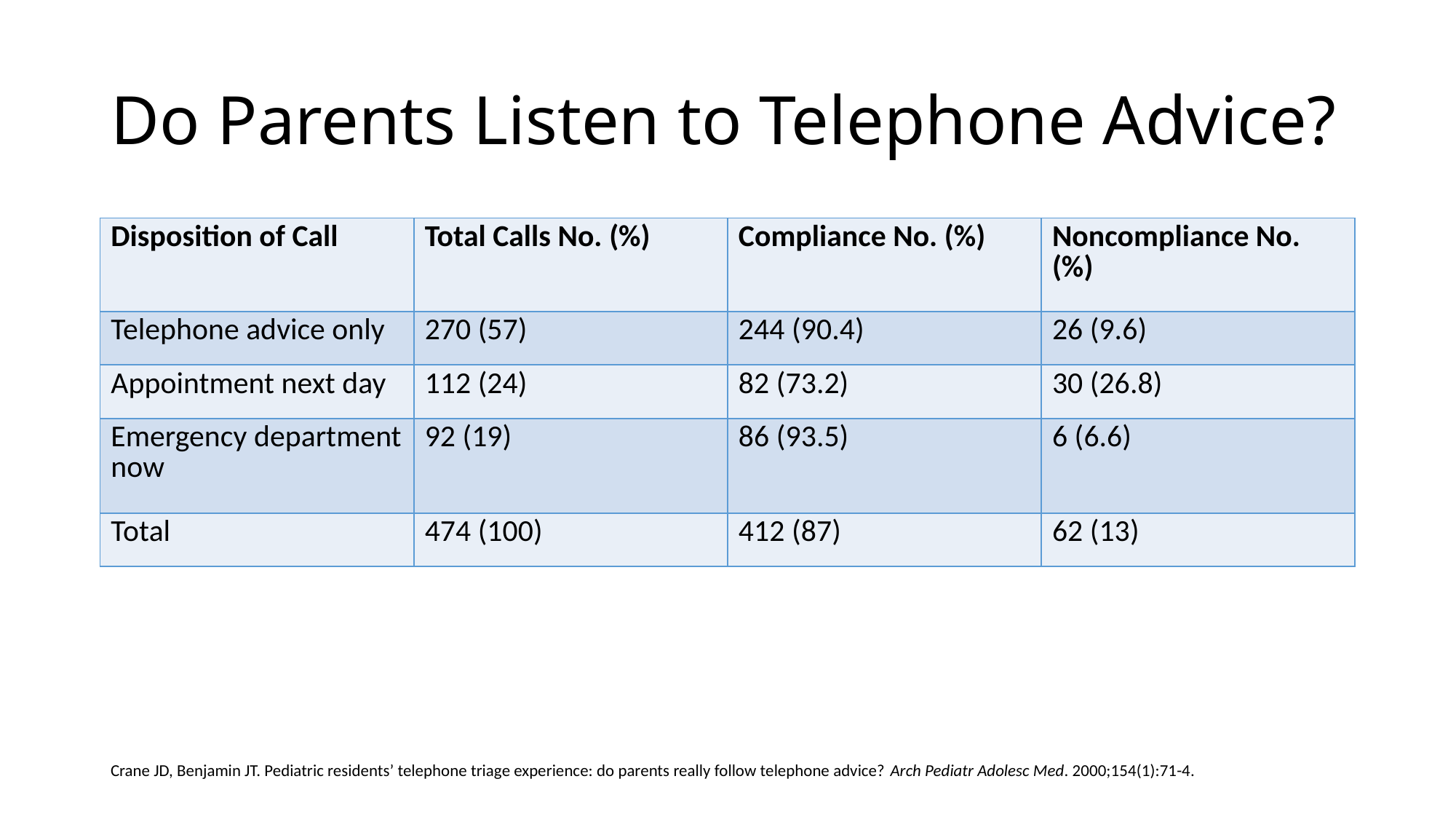

# Do Parents Listen to Telephone Advice?
| Disposition of Call | Total Calls No. (%) | Compliance No. (%) | Noncompliance No. (%) |
| --- | --- | --- | --- |
| Telephone advice only | 270 (57) | 244 (90.4) | 26 (9.6) |
| Appointment next day | 112 (24) | 82 (73.2) | 30 (26.8) |
| Emergency department now | 92 (19) | 86 (93.5) | 6 (6.6) |
| Total | 474 (100) | 412 (87) | 62 (13) |
Crane JD, Benjamin JT. Pediatric residents’ telephone triage experience: do parents really follow telephone advice? Arch Pediatr Adolesc Med. 2000;154(1):71-4.

## Slide 9
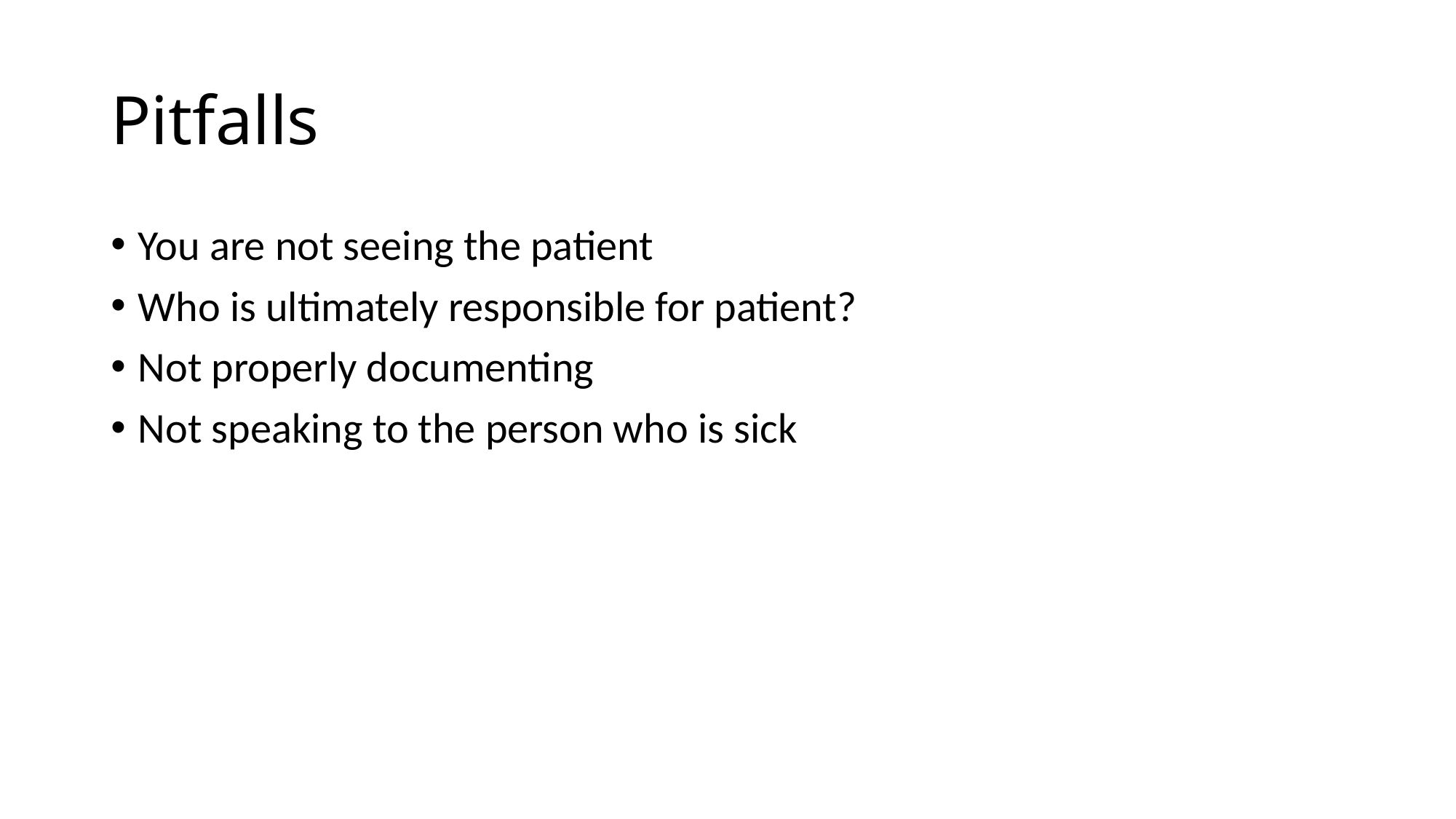

# Pitfalls
You are not seeing the patient
Who is ultimately responsible for patient?
Not properly documenting
Not speaking to the person who is sick

## Slide 10
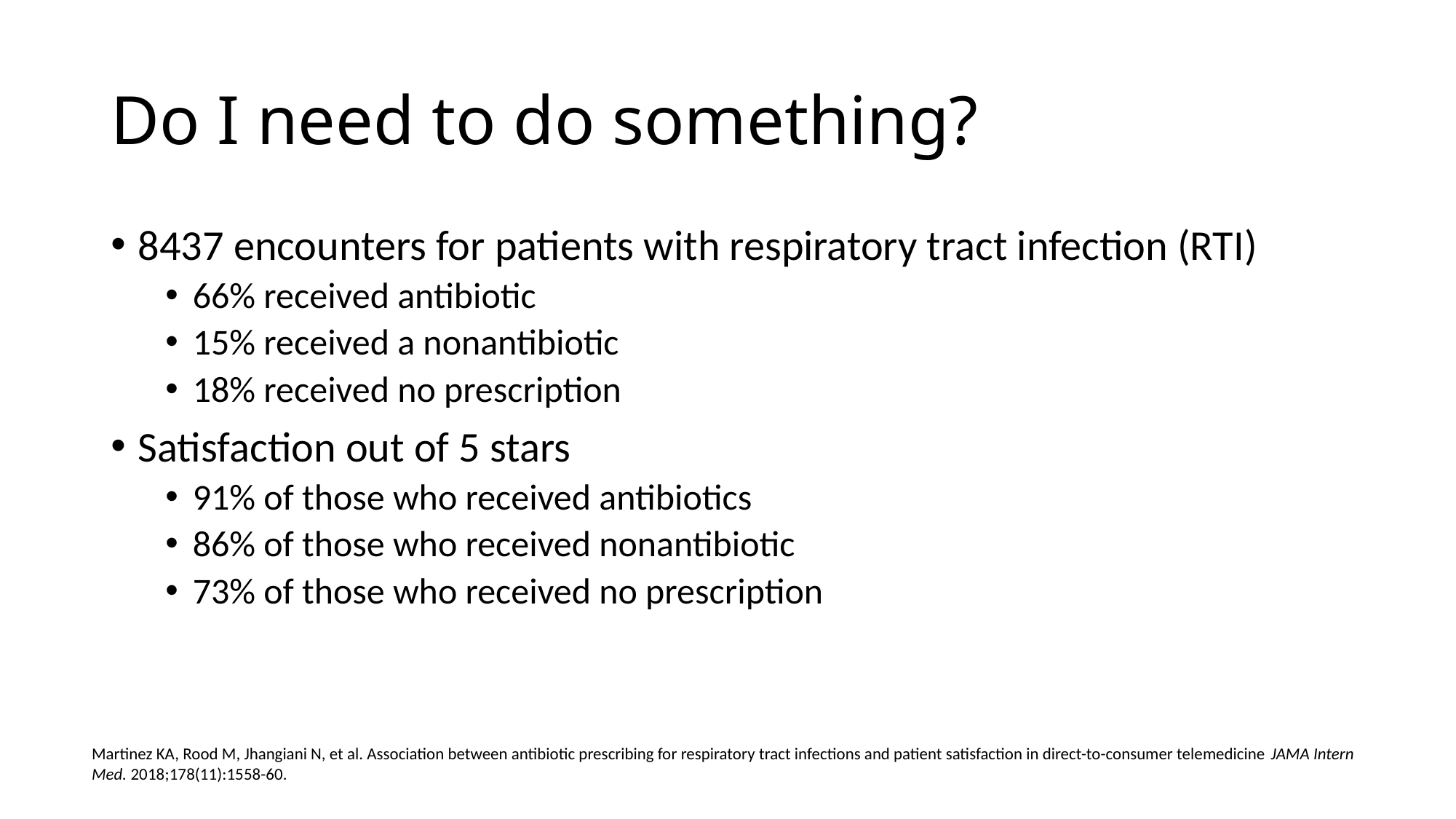

# Do I need to do something?
8437 encounters for patients with respiratory tract infection (RTI)
66% received antibiotic
15% received a nonantibiotic
18% received no prescription
Satisfaction out of 5 stars
91% of those who received antibiotics
86% of those who received nonantibiotic
73% of those who received no prescription
Martinez KA, Rood M, Jhangiani N, et al. Association between antibiotic prescribing for respiratory tract infections and patient satisfaction in direct-to-consumer telemedicine JAMA Intern Med. 2018;178(11):1558-60.

## Slide 11
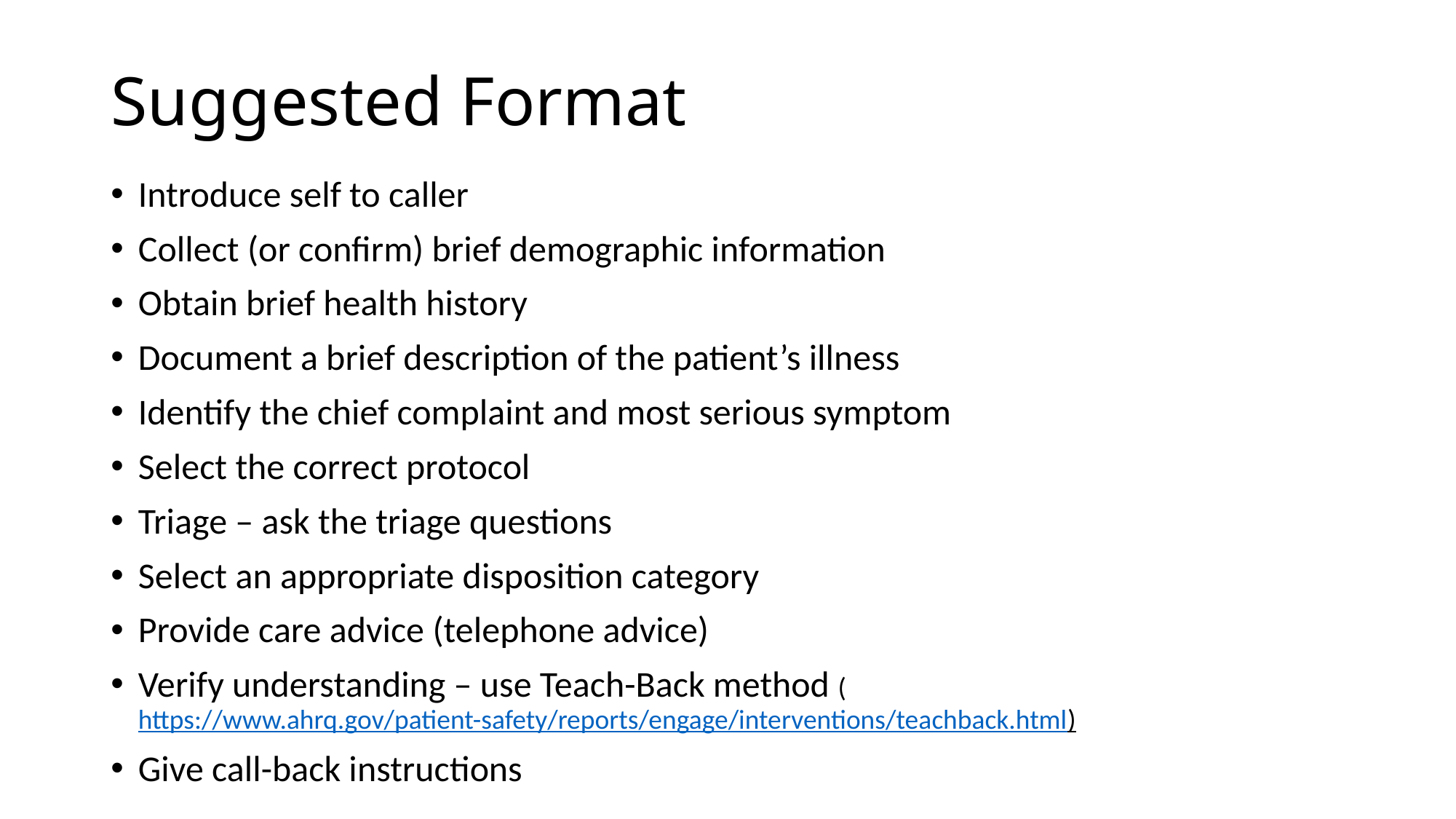

# Suggested Format
Introduce self to caller
Collect (or confirm) brief demographic information
Obtain brief health history
Document a brief description of the patient’s illness
Identify the chief complaint and most serious symptom
Select the correct protocol
Triage – ask the triage questions
Select an appropriate disposition category
Provide care advice (telephone advice)
Verify understanding – use Teach-Back method (https://www.ahrq.gov/patient-safety/reports/engage/interventions/teachback.html)
Give call-back instructions

## Slide 12
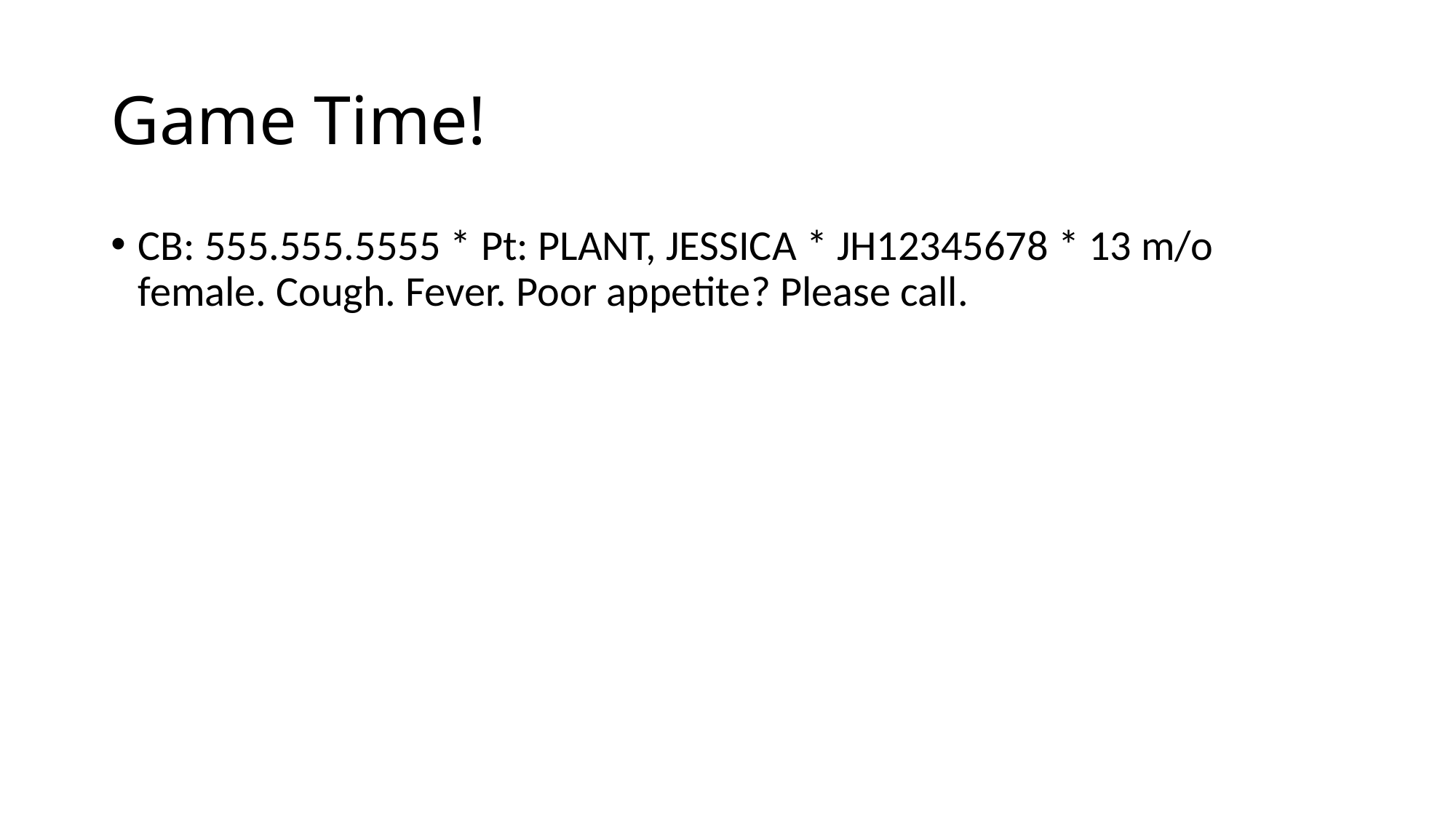

# Game Time!
CB: 555.555.5555 * Pt: PLANT, JESSICA * JH12345678 * 13 m/o female. Cough. Fever. Poor appetite? Please call.
