# Supplementary material for: Phone It In: A Medical Student Primer on Telemedicine Consultation in Pediatrics
Source: MedEdPORTAL. 2021 Jan 7;17:11067. doi: 10.15766/mep_2374-8265.11067 (PMC7809927; doi:10.15766/mep_2374-8265.11067)
Supplement: Supplementary file 1 — Facilitator Guide.docxPhone It In Presentation.pptxSpeaker Notes.docxTelemedicine Cases.docxSession Evaluation.docx [file mep_2374-8265.11067-s001.zip › C. Speaker Notes.docx]

**Speaker Notes for PowerPoint Presentation**

Slide 1: The purpose of today’s session is to introduce you to the benefits, challenges, and mechanics of telephone triage. Proficiency in telehealth technology is rapidly increasing in importance, yet it requires a unique skillset that is often not explicitly taught prior to entry into practice. We have a brief slide set designed to share some of the published data on this topic, but we will spend the majority of the session practicing this skill.

Slide 2: By the end of this session, you will be able to list questions to ask a patient who calls a triage line based on the patient’s chief concern, differentiate severity of illness through phone conversation with a patient or patient’s caregiver, and develop a management plan based on assessment through a phone conversation.

Slide 3: Telephone medicine and triage is not unique to pediatrics. Even prior to the COVID-19 era, nearly a quarter of all patient encounters occurred outside of the traditional office setting. It is important to not only understand the mechanics of telephone triage, but to also be mindful of its limitations to ensure that patient care provided via this modality remains evidence-based and cost-effective. In one study in Pediatrics, children were more likely to receive non-indicated antibiotics when cared for exclusively via the telephone.

Slide 4: One of the most significant limitations to more widespread use of telemedicine technology has been related to reimbursement. In 2018, the introduction of virtual care codes allowed for providers to bill for services provided using technology without participating in an in-person encounter. At the end of April 2020, during the COVID-19 pandemic, CMS further expanded reimbursement for telehealth services, particularly for those provided via the telephone.

Slide 5: When approaching a telephone encounter with a patient or a parent, your primary goal is to determine which of these three paths is most appropriate for your patient: Reassure and manage at home, make a next day appointment, or go to the ED.

Slide 6: A natural follow-up question is how often is our assessment of the severity of a patient’s status wrong (especially considering that the majority of telephone triage lines are first answered by a non-physician provider)? In one study, it was determined that under-referral resulted in adverse patient outcomes in only 1-3% of encounters. Notably, the existence of a telephone triage line decreased unnecessary utilization of the healthcare system, as a vast majority of parents indicated that they would have visited an urgent care, ED, or retail clinic, had telemedicine services not been available to them.

Slide 7: In pediatrics, the most common chief complaints of telephone encounters include those noted here, with fever, cough, and vomiting at the top of the list. You can see from this study that on average 2/3 of these calls are managed with telephone advice only. The remainder of calls resulted in an even distribution between AM appointments and ED visits.

Slide 8: As you can see from this study, over 90% of parents followed the advice they received via the telephone encounter to either remain home or go to the ED. A smaller percentage, 73%, followed the advice to schedule an appointment the next day. This was likely explained by children improving overnight before the appointment and parents subsequently deciding to not bring them into the office.

Slide 9: As you might imagine, there are inherent challenges of telephone triage. Most notably, you are unable to exam the patient in the same way you would in person. In pediatrics especially, you frequently complete the encounter without ever speaking to the person who is ill. Ensuring appropriate follow-up and determining responsibility/liability for patient outcomes can be challenging and is made more complicated without clear established documentation standards.

Slide 10: This study looked at adult patients treated for a respiratory tract infection via the telephone. You can see that the majority of patients received a prescription for an antibiotic, which one could argue was likely an over-prescription of antibiotics considering that these patients were not physically examined. There was also a trend of increased patient satisfaction when a prescription (especially an antibiotic prescription) was provided. That being said, nearly 3/4 of patients who received no prescription were still satisfied with the encounter. You will find patients and parents often call desiring reassurance or guidance on supportive treatment strategies. You should not feel that the only way to resolve a telephone encounter is with a prescription.

Slide 11: Here is a format you can follow when conducting a telephone triage call. The reading you completed prior to today’s session is from a book by Barton Schmidt called Pediatric Telephone Protocols. You can reference texts such as this to help you guide triage of specific diagnoses. When completing a telephone triage call, you will not need to collect as extensive of a history as you would in a traditional H&P. You should aim to ask focused questions to help you determine the severity of the patient’s illness. You do not necessarily need to definitively diagnose the patient either, especially if you determine they need urgent, in-person evaluation. Whatever advice you give the parent or patient, it is important to ensure that they understand your instructions. The Teach-Back method can be helpful here. Regardless of your disposition decision, it is crucial to give instructions about symptoms or events that would warrant either a call-back or an escalation of care.

Slide 12: Now it’s time to practice! You are the physician on call for a large outpatient pediatrics practice and you receive the following page from your practice’s central triage line. We will need a volunteer to come to the front and return this parent’s call. Your colleagues can help with questions and you can take time-outs to discuss the case as a group whenever you would like.
