# Supplementary material for: Phone It In: A Medical Student Primer on Telemedicine Consultation in Pediatrics
Source: MedEdPORTAL. 2021 Jan 7;17:11067. doi: 10.15766/mep_2374-8265.11067 (PMC7809927; doi:10.15766/mep_2374-8265.11067)
Supplement: Supplementary file 1 — Facilitator Guide.docxPhone It In Presentation.pptxSpeaker Notes.docxTelemedicine Cases.docxSession Evaluation.docx [file mep_2374-8265.11067-s001.zip › D. Telemedicine Cases.docx]

**Telemedicine Cases**:

**Case 1:**

**History of Present Illness**: 13-month-old previously healthy girl with cough, fever, and poor appetite. Since yesterday afternoon, she has been crying more than usual, has had a runny nose and cough, and feels warm (parents do not have a thermometer). She does not seem to be as hungry as usual but continues to drink normally. She has made 4 wet diapers since yesterday afternoon.

**Birth History**: Born at 41 weeks via SVD. APGARS 8, 9. Discharged home at 2 days of life.

**Past Medical/Surgical History**: Gastroesophageal reflux disease

**Medications:** Famotidine

**Allergies**: NKDA

**Social History**: Lives with mom and dad. Paternal grandmother watches the baby during the day. She has no sick contacts but does have an older brother that goes to day care.

**Immunizations**: Vaccines are UTD.

**Family History**: Aunt with celiac disease. Maternal grandmother had "a heart condition" in her old age. Mother healthy.

**Notable Exam Findings (for the reference of the facilitator)**:

**Vital Signs**: She is febrile to 38.4, breathing at a rate of 25 breaths per minute, has a normal heart rate of 100, and has a normal oxygen saturation on room air.

**Eyes and Nose:** She is making tears and has moist mucus membranes. She has rhinorrhea and upper airway congestion.

**Neck**: Her neck is supple.

**Heart and Lungs**: She has no signs of increased work of breathing – no nasal flaring, no grunting, no head bobbing, no accessory muscle use. She has normal pulses and brisk capillary refill. If she were to have an auscultatory exam, she would have clear lung sounds and normal heart sounds with a normal rate and rhythm and without a murmur.

**Abdomen**: She has a normal, soft, non-tender abdominal exam.

**Neurologic:** She is fussy but has a normal mental status and normal tone.

**Skin**: She has no rashes.

**Case 2**:

**History of Present Illness**: 13-month-old previously healthy girl with cough, fever, and poor appetite. For the past five days, she has been less interested in eating and drinking. She has been making fewer wet diapers over the past few days and has only made one wet diaper in the past 24 hours. She has a cough and runny nose, which has worsened over the past day. She has been more sleepy than usual and seems to be having trouble breathing. Parents have been unable to take a temperature but note that she is “burning up.”

**Birth History**: Born at 41 weeks via SVD. APGARS 8, 9. Discharged home at 2 days of life.

**Past Medical/Surgical History**: Gastroesophageal reflux disease

**Medications**: Famotidine

**Allergies**: No known drug allergies

**Social History**: Lives with mom and dad. Paternal grandmother watches the baby during the day. She has no sick contacts but does have an older brother that goes to day care.

**Immunizations**: Vaccines are UTD.

**Family History**: Aunt with celiac disease. Maternal grandmother had "a heart condition" in her old age. Mother healthy.

**Notable Exam Findings (for the reference of the facilitator)**:

**Vital Signs**: She is febrile to 38.4, tachycardic to 140, breathing at a rate of 60, and has an oxygen saturation of 85% on room air.

**Eyes and Nose:** She has decreased tear production and dry mucous membranes. She has rhinorrhea and upper airway congestion.

**Neck:** Her neck is supple

**Heart and Lungs**: She is tachypneic to 60 with intercostal, subcostal, and suprasternal retractions. No nasal flaring, grunting, or head bobbing. She has normal pulses and slightly increased capillary refill of 3 seconds. If she were to have an auscultatory exam, she would have crackles in the right lower lung field and would have a tachycardic heart rate with a normal rhythm and no murmur.

**Abdomen:** She has a normal, soft, non-tender abdominal exam.

**Neurologic:** She is fussy but has a normal mental status and normal tone.

**Skin**: She has no rashes.
