# Supplementary material for: Phone It In: A Medical Student Primer on Telemedicine Consultation in Pediatrics
Source: MedEdPORTAL. 2021 Jan 7;17:11067. doi: 10.15766/mep_2374-8265.11067 (PMC7809927; doi:10.15766/mep_2374-8265.11067)
Supplement: Supplementary file 1 — Facilitator Guide.docxPhone It In Presentation.pptxSpeaker Notes.docxTelemedicine Cases.docxSession Evaluation.docx [file mep_2374-8265.11067-s001.zip › E. Session Evaluation.docx]

1. Did the seminar meet the stated objectives?
   1. Not at all
   2. Somewhat
   3. Completely
2. To what extent did the seminar increase your knowledge of the topic?
   1. Not at all
   2. Somewhat
   3. Completely
3. The presenter’s presentation style actively engaged me.
   1. Strongly disagree
   2. Neutral
   3. Strongly agree
4. The session’s overall quality was:
   1. Poor
   2. Average
   3. Excellent
5. Please use the space below to provide us with any comments or suggestions you may have.
